# Characterization and Degradation Behavior of Agar–Carbomer Based Hydrogels for Drug Delivery Applications: Solute Effect

**DOI:** 10.3390/ijms12063394

**Published:** 2011-05-25

**Authors:** Filippo Rossi, Marco Santoro, Tommaso Casalini, Pietro Veglianese, Maurizio Masi, Giuseppe Perale

**Affiliations:** 1 Dipartimento di Chimica, Materiali e Ingegneria Chimica “Giulio Natta”, Politecnico di Milano, Via Mancinelli 7, 20131 Milano, Italy; E-Mails: filippo.rossi@mail.polimi.it (F.R.); marco.santoro@chem.polimi.it (M.S.); tommaso.casalini@mail.polimi.it (T.C.); maurizio.masi@polimi.it (M.M.); 2 Dipartimento di Neuroscienze, Istituto di Ricerche Farmacologiche “Mario Negri”, via La Masa 19, 20156 Milano, Italy; E-Mail: pietro.veglianese@marionegri.it

**Keywords:** degradation, drug effect, FT-IR, hydrogels, rheology

## Abstract

In this study hydrogels synthesized from agarose and carbomer 974P macromers were selected for their potential application in spinal cord injury (SCI) repair strategies following their ability to carry cells and drugs. Indeed, in drug delivery applications, one of the most important issues to be addressed concerns hydrogel ability to provide a finely controlled delivery of loaded drugs, together with a coherent degradation kinetic. Nevertheless, solute effects on drug delivery system are often neglected in the large body of literature, focusing only on the characterization of unloaded matrices. For this reason, in this work, hydrogels were loaded with a chromophoric salt able to mimic, in terms of steric hindrance, many steroids commonly used in SCI repair, and its effects were investigated both from a structural and a rheological point of view, considering the pH-sensitivity of the material. Additionally, degradation chemistry was assessed by means of infrared bond response (FT-IR) and mass loss.

## 1. Introduction

Tissue engineering, briefly the smart combination of life sciences and engineering to replace damaged or missing parts of living tissues, is widely accepted as being the future in regenerative medicine and health care [1–3]. Recent literature suggests a combined strategy of cells and appropriated drugs delivered directly *in situ* as a promising tool to counteract many diseases [4–6]. All these approaches agreed about the use of biomaterials as a device in drug and cell delivery applications, thus answering the typical medical shortcoming of an uncontrolled outwards migration of the delivered product from the target tissue [7,8]. Among the huge field of biomaterials, emerging strategies in regenerative medicine show a very strong interest in hydrogel, being an excellent candidate for combined cell and drug delivery [9–11].

Indeed, hydrogels are formed by high absorbent three-dimensional hydrophobic polymeric networks, which are able to mimic natural living tissues in terms of elasticity and mechanical properties [4,12].

Conventionally, hydrogels may be classified as either synthetic or natural in origin [4]. On one hand, synthetic polymers can be tuned in terms of composition, rate of degradation, mechanical and chemical properties [13,14]. On the other hand, naturally derived polymers provide structures resembling living tissues in terms of specific cellular response, which sometimes overcome the advantages of synthetic polymers [15,16]. Moreover, natural polymers show similarity with the extracellular matrix (ECM), with the advantage of avoiding the stimulation of chronic inflammation, immunological reactions or toxicity, a characteristic drawback of synthetic polymers. However attention must be paid not only to ensure complete biocompatibility of both intermediate and final degradation macromers, but also to provide a degradation kinetic compatible with host tissue integration, in order to allow proper and viable tissue regenerative processes [17,18]. Nevertheless, the presence of drug loaded within the polymeric network is mostly neglected in literature [9,19], with the heavy risk to underestimate its role on material synthesis, characterization and degradation.

The present work deals with controlled drug delivery from a new highly biocompatible and pH-dependent hydrogel [20–23], which was specifically developed for regenerative medicine applications in spinal cord injury (SCI) repair [24,25]. The hydrogel here investigated is also briefly named with the *ACx* acronym, from the two macromers used for their synthesis: agarose and carbomer 974P, reacting in a statistical block polycondensation [21,24]. In general, the polymeric blend can be easily tuned using adequate proportions of cross-linking agents in order to obtain a material library, AC*x* indeed, able to accomplish different drug delivery needs [20,21]. In particular, here the *AC1* hydrogel was investigated as drug carriers for local delivery purposes of Sodium Fluorescein (SF), a good and commonly used drug-mimetic molecule [20,26,27]. In fact, SF shows steric hindrance and molecular structure similar to many corticosteroids and anti-inflammatory drugs (for example methyl prednisone and β-estradiol), which are regularly used in pharmacotherapy to ameliorate SCI secondary effects and central nervous system (CNS) damage [9,19,28].

Its role was examined by means of swelling kinetics, degradation, drug delivery kinetic and rheological properties together with FT-IR analysis. In addition, being the polymeric network anionic and with a high pH-dependence due to the presence of phosphate buffered saline solution (PBS) as solvent in its synthesis, this key parameter was also considered and studied, providing new deeper insights to previous investigations [21,22].

## 2. Results and Discussions

### 2.1. Drug Delivery

**Figure 1:**
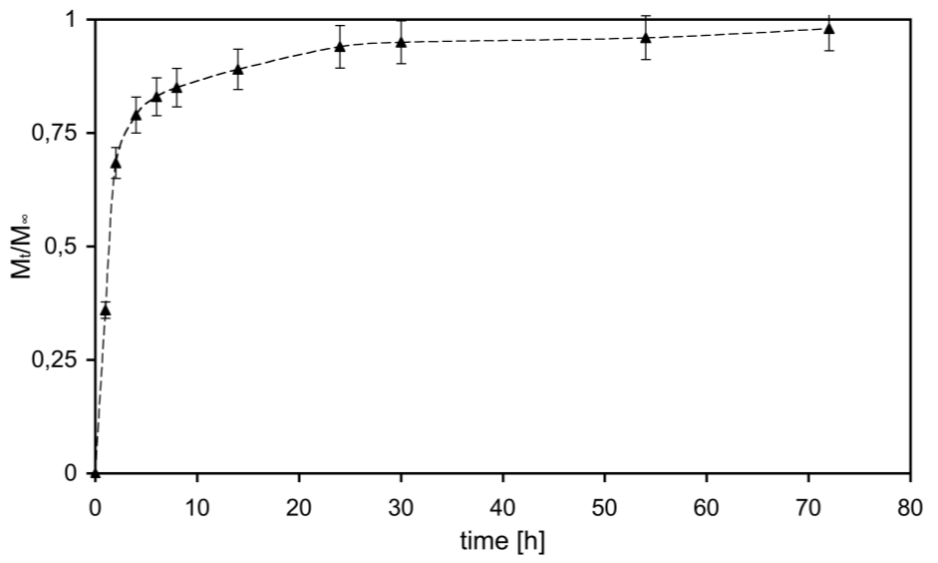
SF release profile from *AC1* (*M*_t_) expressed as unitary fraction with respect to total loaded mass (*M*_∞_).

At first, hydrogel ability to release drugs in accordance with medical needs was investigated and the results are presented in Figure 1, as cumulative mass fraction released in the outer solution (M_t_/M_∞_).

Figure 1 shows rapid initial release of SF due to the initial high concentration gradient leading to a burst effect [29]. This burst could be more likely caused by: (*i*) molecules that were at or near the solvent-hydrogel interface and thus could rapidly escape into the supernatant solution; and (*ii*) molecules that found a fast path through large pores of the hydrogel, with respect of those that had to diffuse through smaller ones, thus partially suffering a constrained molecular motion. After this initial burst, release became slower and reached an almost steady state condition after about 48 h (*plateau*). Moreover, it has to be noted that all loaded SF was released: while this confirms the presence of high clearance, it also gives indirect, but very important, information about the absence of chemical stable interactions between SF and the hydrogel matrix. Furthermore, SF complete release time, about 48 h, is fully compatible with medical needs on steroids like methylprednisolone [9,30,31]. For the above-mentioned considerations it can be assumed that all loaded SF is solved within hydrogel internal medium and is free to diffuse out, driven by concentration gradients. This can easily be explained considering the ratio between mean gel network mesh size (45 nm [20]) and SF mean hydrodynamic *radius* (0.5 nm [27]): diffusant molecules are not physically entrapped inside the entangled hydrogel network and are thus allowed to diffuse with a high free motion [10,20].

### 2.2. Material Characterization

#### 2.2.1. Swelling Behavior

**Figure 2:**
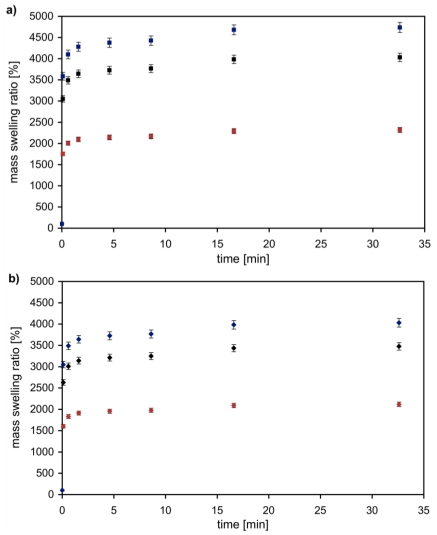
Swelling kinetics of *AC1* **(a)** and *AC1_SF* loaded hydrogel **(b)** in different pH solvent values (pH = 0.5 (red); pH = 7.4 (black); and pH = 14 (blue)).

Hydrogel here investigated was synthesized starting from a high molecular weight branched polyacrylic acid (carbomer 974P) and agarose, a common polysaccharide. The condensation reaction, occurring in PBS solution, was microwave-assisted to obtain a chemically cross-linked hydrogel, as verified with FT-IR analysis and presented in the following section [21]. Generally, chemical interactions would statistically bring polymer chains together and, indeed, the formation of a stable structure occurs through junction zones between chains. The presence of hydroxyl groups and carboxyl ones suggests that main interactions occur via esterification and hydrogen bonding (chemical hydrogel), as confirmed by FT-IR analysis. Moreover, PBS salts freely solvated in water cause salt carboxylates formation. Due to these reactions, *AC* hydrogels are quite anionic and this electrostatic nature, confirmed by mass equilibrium swelling at different pH, influences the ability and the kinetics involved in drug delivery [20]. The ability to absorb and retain a large amount of water is the key feature for 3D polymeric networks, such as hydrogel systems: swelling is thus the first point to investigate. Figure 2 shows the swelling kinetics of unloaded (Figure 2a) and loaded (Figure 2b) *AC1* hydrogel at 37 °C and 5% CO_2_, at different pH values. All samples exhibited fast swelling kinetics and they reached swelling equilibrium within the first hour. On one side the dependence on pH is well visible in Figure 2a: the ability to retain water increases together with solvent pH. A possible explanation could be that, because of the presence of carbomer (having a pKa value around 6.0 in a pH 7.2 phosphate buffer), the carboxylic groups of carbomer are highly dissociated. Therefore the carboxylate moiety on the polymer ionizes, resulting in repulsion between the negative charges, which favors the swelling of the polymer [32]. Adding positive charges (acid pH values of swelling solvent), negative charged backbones are partially shielded and attractive electrostatic forces rule the overall swelling process, favoring network shrinking. On the other hand, adding negative charges (basic pH values) prevailing forces are the repulsive ones, with a consequent higher stretching degree of the structure. Furthermore, mean mesh size of the polymeric network can be tuned by controlling swelling solvent pH, according to the specific requirements of the employed cells, and with respect to the specific regenerative medicine application [22]. Moreover it is easily observable that swelling phenomenon is also strictly dependent on the solute loaded within the polymeric network. Indeed the same pH dependence is present in loaded samples, but the respective mass swelling equilibrium values are lower compared with samples in Figure 2a.

This effect is probably due to repulsion forces between negatively charged polymer backbones and the molecule loaded. This compression reduces natural repulsive forces between polymer chains, with consequent reduction of the amount of water that can be absorbed during swelling mechanism.

In fact, as shown in Figure 2b solute concentration causes the swelling ratio to decrease, which agrees with the above-mentioned hypothesis of charged species shielding polymer chain’s charges. The presence of the drug, loaded within the network, represents an encumbrance to the swelling phenomenon and consequently the percentage of solvent absorbed decreases.

#### 2.2.2. Fourier Transform Infrared Spectroscopy (FT-IR)

**Figure 3:**
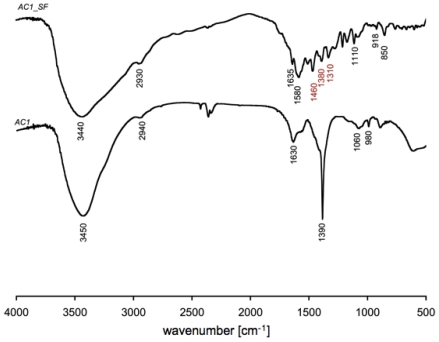
Fourier transform infrared (FT-IR) spectra for both *AC1* and *AC1_SF* gel samples. In red are highlighted peaks showing the presence of SF within the three-dimensional polymeric network.

FT-IR spectra of *AC1* and *AC1_SF* gel are plotted in Figure 3. Both hydrogels spectra show a broad peak around 3450 cm^−1^, which is due to the stretching vibration of O-H bonds, while peaks around 2940 cm^−1^ are due to the C-H stretch. According with our previous studies, the formation of ester bonds is visible in peaks corresponding to symmetric (around 1600 cm^−1^) and asymmetric (around 1400 cm^−1^) CO^2^ stretches. Moreover, the presence of triethylamine (TEA) inside the network, related to C-N vibration, is confirmed by the peaks around 1080 cm^−1^. Spectra also show, in the range 900–1000 cm^−1^, peaks related to C-O-C stretch vibration that represents the glycosidic bond between monosaccharide units (typical of agarose structure) and ester groups.

In general the building blocks, or subunits, of macromolecules form a stable structure made up mostly of C-C bonds, usually referred as the “carbon skeleton”. C-C and C-H bonds are said to be non polar and, thus, tend to be less reactive and sometimes result inert at our degradation conditions. Building blocks of macromolecules act as discrete subunit because their internal structure consists of C-C bonds. Furthermore, C-O and/or C-N bonds make the links between the subunits representing degradable bonds constituted by oxygen or nitrogen atoms. Here, the obtained FT-IR results allow stating that the most important groups in the studied gels are: -OH, C-H, CO_2_ (*i.e.*, carboxylates), vinyl, C-N, and C-O-C. *AC1_SF* FT-IR spectrum showed the presence of sodium fluorescein loaded within the polymeric network by peaks (in red) in correspondence of 1460 and 1380, related to C-C aromatic skeletal stretch and C-H stretches in the aromatic ring, respectively. Furthermore the peak around 1310 cm^−1^ is characteristic of fluorescein dianion, showing the presence of the phenoxide-like stretch [33].

#### 2.2.3. Rheology

**Figure 4:**
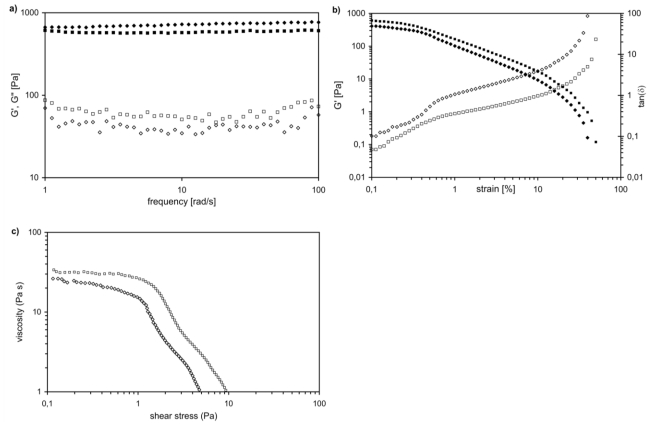
**(a)** Mechanical spectra of *AC1* and *AC1_SF* gels at room temperature with small oscillatory shear in the linear viscoelastic regime: G′ (■ *AC1*, ♦*AC1_SF* ) and G″ (□ *AC1*, ⋄ *AC1_SF* ) are frequency independent, indicating dominant viscoelastic relaxations at lower frequencies; **(b)** Dynamic strain sweep experiments of *AC1* (G′ (■) and tan(*δ*) (□)) and *AC1_SF* (G′ (♦) and tan(*δ*) (⋄)) at constant frequency; **(c)** Measure of yield stress of *AC1* (■) and *AC1_SF* (♦) by oscillatory experiments.

Figure 4a shows the Dynamic Frequency Sweep test (DFS) spectra performed at 37 °C for both loaded (*AC1_SF*) and unloaded (*AC1*) gel samples. In both cases, storage modulus (G′) is approximately one order of magnitude higher than the loss modulus (G″), showing an elastic behavior rather than a viscous one. Moreover, both G′ and G″ of each gel are essentially independent from frequency over the entire investigated range, thus indicating dominant viscoelastic relaxations of networks at lower frequencies. This means that network relaxation time, τ, is rather long (τ ≈ 2000 s) [21]. Such rheological behavior matches the characteristic signature of a solid-like gel, confirming this nature for both loaded and unloaded gels. The values obtained for G′ (~700 Pa for *AC1* and ~580 Pa for *AC1_SF*) shows the same order of magnitude as the modulus reported in literature for other biopolymers and hydrogels. Previous investigations about thixotropic properties of loaded hydrogel samples suggest that hysteresis loop areas are lower: SF molecules indeed make the gels less stable and cause lower mechanical properties. Sol-gel transition is faster and this effect could be a relevant improvement for gel injectability [34]. Indeed, in Figure 4b, the results of the dynamic strain sweep test were shown: for both hydrogel samples, the elastic modulus (G′) dominates at low values of strain and above the cross-over strain (*γ_c_*), the contribution of tan (δ) overwhelms the elastic one. It is also evident that cross-over strain decreases loading solute within the three-dimensional matrix, ranging from 0.30 for *AC1* to 0.1 for *AC1_SF*. The last comparison between the two samples can be done in terms of yield stress (Figure 4c), which decreases from *AC1* to *AC1_SF*.

In summary, the higher material instability makes our hydrogel less stable and able to flow under lower stress values and this is in complete agreement with previous thixotropic studies performed [20].

### 2.3. Hydrogel Degradation

The polymeric matrix could be approximated as a C-C skeleton, due to the presence of carbomer 974P, esterified with agarose and carboxylated by salts present in PBS [21,22]. During the hydrolysis reaction esters become carboxylic acid with the consequent cross-linking loss, following the proposed reaction:

$$RCO_{2}R^{\prime }+H_{2}O\to RCO_{2}H+R^{\prime }OH$$

**Figure 5:**
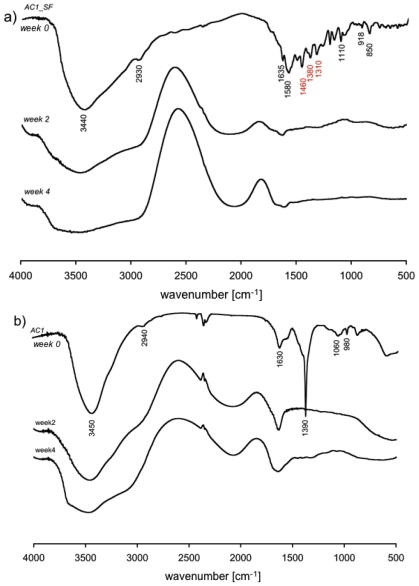
**(a)** FT-IR spectra of *AC1_SF* before and after 2 and 4 weeks of degradation in PBS; **(b)** FT-IR spectra of *AC1* before and after 2 and 4 weeks of degradation in PBS.

Spectra for *AC1* and *AC1_SF* gel before and during hydrolytic degradation are presented in Figure 5.

In both dry sample presented (week 0), the following aspects can be observed: –OH peak stretch (around 3440 cm^−1^), asymmetric C-H stretch (2930 cm^−1^), asymmetric CO_2_ stretch (1580 cm^−1^), symmetric CO_2_ stretch (1390 cm^−1^), asymmetric C-O-C (1080 cm^−1^), C-N bend (1042 cm^−1^), vinyl group (990 cm^−1^) and symmetric C-O-C stretch (892 cm^−1^) plus sodium fluorescein contribution as described above.

After day 1, the complete release of SF from the polymeric matrix (Figure 1) allows us to consider the matrix as unloaded and this is supported by IR spectra (Figure 5a), where SF presence is not detectable confirming the absence of stable bonds between the solute and the matrix.

Moreover, the –OH peak stretch is frequent in this kind of materials because of the large amount of hydroxyl groups and with hydrolytic events its contribution increases and the area thus becomes larger and rounded. Indeed, prolonging the time of exposure to PBS, the –OH peak in the polymer becomes similar to the –OH peak present in a classic FT-IR water spectrum.

The role of apolar bonds could be well explained looking at the C-H stretch peak (2943 cm^−1^): absence of changes during the time means absence of degradation via hydrolysis. Nevertheless, its shape apparently changes during degradation, but the peak maximum remains at the same wave number value, hence confirming that it is not affected by degradation [35]. This peak shape is different because the proximity with the –OH region seems to cover it, this peak actually is not visible in a wet sample. Furthermore, a region between 2250 and 2000 cm^−1^ is easily observable and both present peaks and their area increase during degradation. This wave number region is typically present in water spectrum and increasing during the time shows a reduced macromer concentration decreased due to the material degradation. Next to this last region there are two peaks corresponding to CO_2_ (carboxylates) stretches. Peak maximum, as degradation proceeds, moves toward lower wave numbers, more strongly in hydrogels with short chains, which confirms their faster degradation due to lower hydrophobicity. The peaks corresponding to the SF presence at day 0 and visible in red in Figure 5a are not yet detectable after 2 days. This time corresponds to the *plateau* time for SF release and is represented in Figure 1, highlighting the possibility to deliver completely drugs with steric hindrance similar to SF.

**Figure 6:**
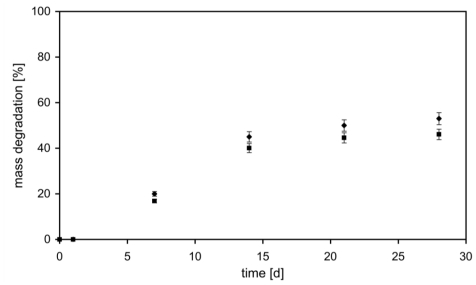
Degradation of *AC1* (■) and *AC1_SF* (♦) hydrogels in PBS at 37 °C with respect to weight loss.

Quantitative consideration on hydrogel degradation was performed as a function of weight loss during incubation time in PBS at 37 °C, as shown in Figure 6. The presence of SF makes our material less stable and the degradation occurs faster. At day 28, the mass degraded of *AC1* and *AC1_SF* hydrogels was 45% and 53%, respectively. On one side, in the first hours the presence of the drug loaded within the network represents an encumbrance to the swelling phenomenon as explained above. On the other, after 48 h SF was completely released (Figure 1) and the resulting unloaded material is less stable respect to *AC1*: the presence of repulsion forces between negatively charged polymer backbones and the molecule loaded creates consequent higher average distances between two following cross-links. So, the degradation phenomenon starts earlier with consequent higher percentage of mass degraded.

## 3. Experimental Studies

### 3.1. Materials

Carbomer 974P, with a molecular weight of about 1 MDa, was provided by Fagron (Rotterdam, The Netherlands), tryethylamine was purchased by Fluka (Buchs, Switzerland). The solvent used was PBS (Phosphate Buffer Saline), purchased by Sigma-Aldrich (Taufkirchen, Germany). As said, the other polymer involved in the reaction is agarose, purchased by Invitrogen (Carlsbad, CA, USA) and having a molecular weight of about 300 kDa. Lastly, SF was provided by Sigma-Aldrich (Taufkirchen, Germany). All materials were used as received.

### 3.2. Hydrogel Synthesis

Carbomer 974P (0.5% w/v) was mixed together with PBS and the resulting solution was neutralized with triethylamine (TEA). Agarose (0.5% w/v) was subsequently added and the system was electromagnetically heated up to 80 °C to induce condensation reactions to begin. The mixture was subsequently merged with a water-based solution (at a 50/50 volumetric ratio), placed in steel cylinders (0.5 mL each and with the same dimensions of a standard well in a 48-plate d = 1.1 cm) and left to rest at 37 °C until reaching complete gelation and thermal equilibrium [21,23,24]. Briefly, in the unirradiated solution polymer chains are not overlapped and thus segmental mobility is high. At low irradiation doses, intramolecular links and chain scissions are privileged. The decrease segmental mobility allows intermolecular cross-links to be formed and thus gives origin to local 3D networks, also known as “microgels”. Increasing irradiation, intermolecular cross-linking and chain scission are massively privileged, thus giving origin to macroscopic gels. Nevertheless, cross-linked gels are not truly homogeneous because clusters of molecular entanglements, hydrophobically domains or ionically-associated ones can create local heterogeneities. Chemical interactions would statistically bring polymer chains together and, indeed, the formation of a stable structure occurs through junction zones between chains [24].

### 3.3. Drug Loading

As said, SF was chosen mainly because of its steric hindrance (MW = 376 Da), similar to many small drugs (e.g., steroids), and for its strong absorbance that makes it easily detectable by UV spectroscopy [20]. Being SF neither temperature sensitive nor interacting with the matrix, loading before gelation was applied: a SF water solution was added to the polymeric formulate while still at sol stage and then the final mixture was left to jellify, as described above. At the end of gelation process SF molecules remain solvated into water, entrapped in the “cavities” of the hydrogel. In order to avoid gel dilution with respect to proportions given, it has to be underlined that SF water solution was used instead of the standard water described in the previous paragraph, keeping the same ratio with respect to the PBS based polymeric solution, that is, 50/50 v/v. SF concentration studied was 0.2 mg/mL, extremely suitable for drug delivery tests [20].

### 3.4. Release Experiments

Before running release experiments, in order to avoid any interference on mass-transfer processes due to swelling phenomena, hydrogel samples were let to swell until equilibrium in isoconcentrated SF aqueous solution overnight [20]. Afterwards, every well was filled with an extreme excess of PBS (keeping pH = 7.4 constant) and stored at 37 °C in a 5% CO_2_ atmosphere. Minimal aliquots were collected at defined time points, while solution was refreshed, in order to avoid attainment of mass-transfer equilibrium between the gel and the surrounding PBS solution and, thus, letting high the concentration gradient driving force [20]. SF concentration was measured by UV spectroscopy applying Lambert-Beer method (λ = 470–490 nm).

### 3.5. Physical Characterization

#### 3.5.1 Swelling Behavior

The hydrogel samples were first immersed in PBS for about 24 h, then freeze-dried, weighted (*W_d_*) and poured in excess solvent at pH = 0.5, 7.4 and 14 to achieve complete swelling at 37 °C in 5% CO_2_ atmosphere; such conditions were applied since they are typical of *in vitro* experiments [21,36]. The swelling kinetics was measured gravimetrically. The samples were removed from the solvent at regular times. Then, hydrogel surfaces were wiped with moistened filter paper in order to remove the excess of solvent and then weighed (*W_t_*) [36,37]. Swelling ratio is defined as follows:

(1) $$swelling\;ratio=\frac{W_{d}(t)}{W_{d}(0)}\times 100$$

where *W_d_(t)* is the weight of the wet hydrogel as a function of time and *W_d_(0)* of the dry one.

#### 3.5.2. FTIR Spectra

Hydrogel samples, after being left dipped for 24 h in excess of solvent, were freeze-dried and laminated with potassium bromide. Infrared spectra were then recorded using a TENSOR Series FTIR Spectrometer (Bruker, Bremen, Germany). FT-IR spectra were recorded using a Thermo Nexus 6700 spectrometer coupled to a Thermo Nicolet Continuum microscope equipped with a × 15 Reflachromat Cassegrain objective.

#### 3.5.3. Rheological Measurements

Rheological analysis on gel samples were performed at 37 °C using a Rheometric Scientific ARES (TA Instruments, New Castle, DE, USA) equipped with parallel plates of 30 mm of diameter and a 4 mm gap between [21]. A human-body like temperature of 37 °C was selected because investigated materials contain substantial amounts of water and higher temperatures could significantly affect their properties, especially long-term ones. Oscillatory responses (*G′*, elastic modulus, and *G″*, loss/viscous modulus) were determined at low strain values (0.02%) over the frequency range 0.1–500 rad/s. Dynamic Strain Sweep tests were also performed at frequency of 20 rad/s over the strain range from 0.01% to 100%. Thixotropic behavior was also investigated and shear strain and viscosity as a function of shear rate were evaluated, too [20]. Linearity of all viscoelastic properties was assessed for all samples.

### 3.6. Degradation

#### 3.6.1. FT-IR Analysis

Hydrogel samples were first freeze dried for about 24 h, then immersed in excess of PBS to swell and then degrade, always being kept at 37 °C in a 5% CO_2_ atmosphere. Samples were removed from PBS (after 1, 2, 3, 7, 14, 21 and 28 days) and compared with dry samples, *i.e.*, those that were freeze-dried but not immersed in PBS. All these samples were analyzed using FT-IR instrument collecting the transmittance signal [21,35].

#### 3.6.2. Mass Loss Kinetic

Degradations were performed in PBS at 37 °C under agitation. The buffer was changed daily, and at 1, 4, 7, 14 and 28 day of incubation the buffer was removed, the polymer samples were lyophilized and degradation was quantified using the following equation [37]:

(2) $$mass\;\text{deg}\;radation=\frac{W_{d}(t)}{W_{d}(0)}\times 100$$

where *W_d_(0)* is the initially dry hydrogel mass and *W_d_(t)* is the dry hydrogel mass at time t.

### 3.7. Statistical Analysis

Where applicable, experimental data were analyzed using Analysis of Variance (ANOVA). Statistical significance was set to p value < 0.05. Results are presented as mean ± SD [17].

## 4. Conclusions

The influence of solute presence within hydrogel polymeric network was studied in order to develop a better understanding of diffusive phenomena and thus to obtain a better controlled drug delivery system, matching the needs of applications into central nervous system. Solutes role is usually neglected during development and engineering of drug delivery devices, mainly because of the extremely low concentrations used and the small steric hindrance of diffusants. Here, agar–carbomer based hydrogels were loaded with sodium fluorescein, a common drug mimetic, performing studies on material characterization, drug delivery kinetics and degradation. The complete release from such devices suggested the absence of stable chemical bonds between the matrix and solute. Moreover, solute role was clearly visible considering swelling kinetics, rheology and degradation profile: the solute presence indeed made our hydrogel less stable, able to flow under lower stress values and more easily degradable.

## References

[1] Langer R, Vacanti JP (1993). Tissue engineering. Science.

[2] Sakurada K, McDonald FM, Shimada F (2008). Regenerative medicine and stem cell based drug discovery. Angew. Chem. Int. Edit.

[3] Atala R, Langer R, Thomson J, Nerem R (2008). Principles of Regenerative Medicine.

[4] Shoichet MS (2010). Polymer scaffolds for biomaterials applications. Macromolecules.

[5] Engel E, Michiardi A, Navarro M, Lacroix D, Planell JA (2008). Nanotechnology in regenerative medicine: The materials side. Trends Biotech.

[6] Langer R (2009). Perspectives and challenges in tissue engineering and regenerative medicine. Adv. Mater.

[7] Ye Z, Zhou Y, Cai H, Tan W (2011). Myocardial regeneration: Roles of stem cells and hydrogels. Adv Drug Deliv Rev.

[8] Mei Y, Saha K, Bogatyrev SR, Yang J, Hook AL, Kalcioglu ZI, Cho SW, Mitalipova M, Pyzocha N, Rojas F, van Vliet KJ, Davies MC, Alexander MR, Langer R, Jaenish R, Anderson DG (2010). Combinatorial development of biomaterials for clonal growth of human pluripotent stem cells. Nature Mater.

[9] Kim YT, Caldwell JM, Bellamkonda RV (2009). Nanoparticle-mediated local delivery of methylprednisolone after spinal cord injury. Biomaterials.

[10] Baumann MD, Kang CE, Stanwick JC, Wang YF, Kim H, Lapitsky Y, Shoichet MS (2009). An injectable drug delivery platform for sustained combination therapy. J. Control. Release.

[11] Baumann MD, Kang CE, Tator CH, Shoichet MS (2010). Intrathecal delivery of a polymeric nanocomposite hydrogel after spinal cord injury. Biomaterials.

[12] Slaughter BV, Khurshid SS, Fisher OZ, Khademhosseini A, Peppas NA (2009). Hydrogels in regenerative medicine. Adv. Mater.

[13] Malafaya PB, Silva GA, Reis RL (2007). Natural-origin polymers as carriers and scaffolds for biomolecules and cell delivery in tissue engineering applications. Adv. Drug Deliv. Rev.

[14] Mano JF, Silva GA, Azevedo HS, Malafaya PB, Sousa RA, Silva SS, Boesel LF, Oliveira JM, Santos TC, Marques AP, Neves NM, Reis RL (2007). Natural origin biodegradable systems in tissue engineering and regenerative medicine: Present status and some moving trends. J. R. Soc. Interface.

[15] Hejcl A, Sedy J, Kapcalova M, Toro DA, Amemori T, Lesny P, Likavcanova-Masinova K, Krumbholcova E, Pradny M, Michalek J, Burian M, Hajek M, Jendelova P, Sykova E (2010). HPMA-RGD hydrogels seeded with mesenchymal stem cells improve functional outcome in chronic spinal cord injury. Stem Cells Dev.

[16] Tabata Y (2009). Biomaterial technology for tissue engineering applications. J. R. Soc. Interface.

[17] Tan HP, Chu CR, Payne KA, Marra KG (2009). Injectable *in situ* forming biodegradable chitosan-hyaluronic acid based hydrogels for cartilage tissue engineering. Biomaterials.

[18] Hoare TR, Kohane DS (2008). Hydrogels in drug delivery: Progress and challenges. Polymer.

[19] Pritchard CD, O’Shea TM, Siegwart DJ, Calo E, Anderson DG, Reynolds FM, Thomas JA, Slotkin JR, Woodard EJ, Langer R (2011). An injectable thiol-acrylate poly(ethylene glycol) hydrogel for sustained release of methylprednisolone sodium succinate. Biomaterials.

[20] Santoro M, Marchetti P, Rossi F, Perale G, Castiglione F, Mele A, Masi M (2011). A smart approach to evaluate drug diffusivity in agar-carbomer hydrogels for drug delivery. J. Phys. Chem. B.

[21] Rossi F, Chatzistavrou X, Perale G, Boccaccini AR (2011). Synthesis and degradation of agar–carbomer based hydrogels for tissue engineering appliactions. J Appl Polym Sci.

[22] Rossi F, Perale G, Masi M (2010). Biological buffered saline solution as solvent in agar–carbomer hydrogel synthesis. Chem. Pap.

[23] Perale G, Giordano C, Bianco F, Rossi F, Daniele F, Tunesi M, Crivelli F, Matteoli M, Masi M (2011). Hydrogel for cell housing in the brain and in the spinal cord. Int. J. Artif. Organs.

[24] Perale G, Veglianese P, Rossi F, Peviani M, Santoro M, Llupi D, Micotti E, Forloni G, Masi M (2011). *In situ* agar–carbomer polycondensation: A chemical approach to regenerative medicine. Mater. Lett.

[25] Perale G, Rossi F, Sundstrom E, Bacchiega S, Masi M, Forloni G, Veglianese P (2011). Hydrogels in spinal cord injury repair strategies. ACS Chem Neurosci.

[26] Perale G, Arosio P, Moscatelli D, Barri V, Muller M, Maccagnan S, Masi M (2009). A new model of resorbable device degradation and drug release: Transient 1-dimension diffusional model. J. Control. Release.

[27] Singh TRR, Woolfson AD, Donnelly RF (2010). Investigation of solute permeation across hydrogels composed of poly(methyl vinyl ether-co-maleic acid) and poly(ethylene glycol). J. Pharm. Pharmacol.

[28] Rodriguez-Tenreiro C, Alvarez-Lorenzo C, Rodriguez-Perez A, Concheiro A, Torres-Labandeira JJ (2007). Estradiol sustained release from high affinity cyclodextrin hydrogels. Eur. J. Pharm. Biopharm.

[29] Koutsopoulos S, Unsworth LD, Nagaia Y, Zhang SG (2009). Controlled release of functional proteins through designer self-assembling peptide nanofiber hydrogel scaffold. Proc. Natl. Acad. Sci. USA.

[30] Bracken MB, Shepard MJ, Collins WF, Holford TR, Young W, Piepmeier J, Leosummers L, Baskin DS, Eisenberg HM, Flamm E, Marshall LF, Maroon J, Wilberger J, Perot PL, Sonntag VKH, Wagner FC, Winn HR (1990). A randomized, controlled trial of methylprednisolone or naloxone in the treatment of acute spinal-cord injury. New Engl. J. Med.

[31] Cao K, Huang L, Liu JW, An H, Shu Y, Han ZM (2010). Inhibitory effects of high-dose methylprednisolone on bacterial translocation from gut and endotoxin release following acute spinal cord injury-induced paraplegia in rats. Neur. Regen. Res.

[32] Wen H, Park K (2010). Oral Controlled Release Formulation Design and Drug Delivery: Theory to Practice.

[33] Wang L, Roitberg A, Meuse C, Gaigalas AK (2001). Raman and FTIR spectroscopies of fluorescein in solutions. Spectrochim. Acta A.

[34] Barbucci R, Pasqui D, Favaloro R, Panariello G (2008). A thixotropic hydrogel from chemically cross-linked guar gum: Synthesis, characterization and rheological behaviour. Carbohydr. Res.

[35] Vidovic E, Klee D, Hocker H (2009). Degradation behavior of hydrogels from poly(vinyl Alcohol)- graft-[poly(rac-lactide)/Poly(rac-lactide-co-glycolide)]: Influence of the structure and composition on the material’s stability. J. Appl. Polym. Sci.

[36] Begam T, Tomar RS, Nagpal AK, Singhal R (2004). Synthesis of poly(acrylamide-co-methyl methacrylate-co-vinyl amine-co-acrylic acid) hydrogels by Hoffman degradation and their interactions with acetaminophen. J. Appl. Polym. Sci.

[37] Gupta D, Tator CH, Shoichet MS (2006). Fast-gelling injectable blend of hyaluronan and methylcellulose for intrathecal, localized delivery to the injured spinal cord. Biomaterials.

